# Germplasm variability-assisted near infrared reflectance spectroscopy chemometrics to develop multi-trait robust prediction models in rice

**DOI:** 10.3389/fnut.2022.946255

**Published:** 2022-08-04

**Authors:** Racheal John, Rakesh Bhardwaj, Christine Jeyaseelan, Haritha Bollinedi, Neha Singh, G. D. Harish, Rakesh Singh, Dhrub Jyoti Nath, Mamta Arya, Deepak Sharma, Satyapal Singh, Joseph John K, M. Latha, Jai Chand Rana, Sudhir Pal Ahlawat, Ashok Kumar

**Affiliations:** ^1^Amity Institute of Applied Sciences, Amity University, Noida, India; ^2^Indian Council of Agricultural Research- National Bureau of Plant Genetic Resources (ICAR-NBPGR), New Delhi, India; ^3^Indian Council of Agricultural Research-Indian Agricultural Research Institute (ICAR-IARI), New Delhi, India; ^4^Indian Council of Agricultural Research-National Bureau of Plant Genetic Resources-Regional Station (ICAR-NBPGR-RS)-Barapani, Barapani, India; ^5^Department of Soil Science, Assam Agricultural University, Jorhat, India; ^6^ICAR-NBPGR-RS-Bhowali, Bhowali, India; ^7^Indira Gandhi Krishi Vishwavidyalaya, Raipur, Chhattisgarh, India; ^8^ICAR-NBPGR-RS-Thrishur, Thrissur, India; ^9^Bioversity International – India Office, New Delhi, India

**Keywords:** NIRS assisted stratified sampling, normal distribution, derivatives and gaps, calibration, validation, brown rice

## Abstract

Rice is a major staple food across the world in which wide variations in nutrient composition are reported. Rice improvement programs need germplasm accessions with extreme values for any nutritional trait. Near infrared reflectance spectroscopy (NIRS) uses electromagnetic radiations in the NIR region to rapidly measure the biochemical composition of food and agricultural products. NIRS prediction models provide a rapid assessment tool but their applicability is limited by the sample diversity, used for developing them. NIRS spectral variability was used to select a diverse sample set of 180 accessions, and reference data were generated using association of analytical chemists and standard methods. Different spectral pre-processing (up to fourth-order derivatization), scatter corrections (SNV-DT, MSC), and regression methods (partial least square, modified partial least square, and principle component regression) were employed for each trait. Best-fit models for total protein, starch, amylose, dietary fiber, and oil content were selected based on high RSQ, RPD with low SEP(C) in external validation. All the prediction models had ratio of prediction to deviation (RPD) > 2 amongst which the best models were obtained for dietary fiber and protein with *R*^2^ = 0.945 and 0.917, SEP(C) = 0.069 and 0.329, and RPD = 3.62 and 3.46. A paired sample *t*-test at a 95% confidence interval was performed to ensure that the difference in predicted and laboratory values was non-significant.

## Introduction

Rice (*Oryza sativa*) is the major staple food for nearly 50% of population of the world, primarily in Asia and Africa, where it is highly cultivated and consumed. Particularly, south-eastern Asian countries have a heavy dependence on rice, while trends in Africa also reflect a continuous increase in rice consumption. In 2019, globally, 755,473,800 tonnes of rice were produced of which nearly 90% (677,276,789 tonnes) were produced in Asia, where major producers from China (211,405,211 tonnes) and India (177,645,000 tonnes) together contributed to nearly 50% of world rice production (1). In India, it is grown in more than one-fifth of the total gross cropped area (43,388,000 hectares) and contributes to 689 kcal/capita per day of food supply.

Rice is a significant contributor of food and nutrient to a major population of the world and is therefore used in the preparations of different culinary with wider applications in food industry than in any other grain. The massive consumption of rice as a major staple food has shown a strong association with high incidences of diabetes, protein–energy malnutrition, and deficiencies for iron, iodine, and vitamin A (2). Out of 10 countries having the largest diabetic population, rice is a major staple food in six of them (3). Amylose, fat, and fiber contents influence the glycemic response that makes it essential to identify rice with high protein, fat, and dietary fiber with different levels of amylose content (AC;^4^). Enrichment of rice for protein and limiting glycemic index would have a significant impact on major health challenges of rice-eating population.

World over efforts are being made to improve the quality of major staple foods, as that can have a major impact on improving the nutritional status of vulnerable people, who draw major calorie needs from them. Healthy rice is suggested to have an increased proportion of dietary fiber, amylose, phospholipids, and protein (5). Diversity in nutrient composition in rice germplasm collections can play an important role in selecting nutri-dense varieties with utility for different food formulations. However, global germplasm collections for rice are huge where International Rice Research Institute alone maintains more than 132,000 accessions. Thus, conventional methods of estimation for different nutrients are not appropriate for evaluating them as they not only require very high input cost for laboratory instrumentation, reagents and chemicals, technically skilled analyst, and high consumption of power but are also highly time-consuming.

Near infrared reflectance spectroscopy (NIRS) is a widely used technique for the non-destructive, fast, and robust analysis of various biomolecules through prediction modeling (such as protein, fat, starch, dietary fiber, fatty acids, amino acids, glucosides, carotenoids, and cyanides) in different food matrices such as meat, fruits, vegetables, grains, and flours (6). Several NIRS-based prediction models have been reported in rice for protein content (PC), AC, fat content, flavonoids, total soluble phenols, antioxidants, and dietary fiber (7–13). These models work well for commercial varieties and market samples but are not suitable for screening germplasm collections where the range of variability is very high. Further, germplasm accessions with extreme value act as a gene source for any trait which is vital for crop improvement programs. National gene bank at ICAR-NBPGR has a total of 106,557 (as per 25 June 2022) rice accessions and thus robust prediction models bearing applicability over extreme ranges are required for screening a large germplasm collection of rice.

Models developed on normally distributed data get more learning from middle-range values and fail in performance when tested with extreme value samples. Hence, it is important to follow a sample selection method to achieve uniform distribution frequency in the entire range of variability for each trait (14). Sample selection based on variations in NIR spectral data by the use of statistical methods like Hierarchical cluster analysis (HCA) enables the grouping of similar accessions (15). Selection of samples from cluster/sub-cluster centers and extreme boundaries provide a diverse set with increased frequency for extreme value samples.

Spectral pre-processing is often employed for removing light scattering effects using techniques such as derivatization (feature extraction), standard normal variate and detrend (SNV-DT), multiplicative scatter correction (MSC), and weighted and inverse MSC (16). Spectral derivatization at multiple levels enhances weak regions of spectra and decodes hidden information (17). Prediction model applicability to get near accurate and precise values also depends on the selection of responsive regions by binning (gap intervals) and noise reduction/smoothening by taking moving average (18).

Processed and standardized NIR spectra contain multiple variables in the form of reflectance that is regressed with targeted traits. Multivariate regression techniques such as partial least square (PLS), principal component regression (PCR), and multiple linear regression are used to generate robust and effective models. Modified PLS (MPLS) is commonly used in NIR modeling and is considered stable and less prone to over fitting due to the influence of intragroup variations (19).

In this study, we have tried to address the limitations posed by normal distribution through NIR spectral-based sample selection for reference analysis and used a combination of pre-processing (derivatives, gap, and smoothening), scatter correction methods, and multivariate regression techniques with the objectives of developing robust prediction models for estimating total protein, starch, amylose, dietary fiber, and oil. Based on hierarchical clustering/sub-clustering of NIR spectral data, our strategy of selecting samples helped in achieving a highly diverse calibration set, and the developed models also performed well for extreme values. These models have the applicability for multiple sectors that include gene banks, food and seed industry, and plant breeders working on improving the nutritional value of rice varieties.

## Materials and methods

### Sample preparation

Almost 500 accessions of rice landraces were collected from different states of India representing North Eastern Himalaya, Eastern Himalaya, Northern plains, Central India, and Southern India to accommodate variability in rice landraces due to evolution in specific niche areas. All paddy samples were oven dried at 60^°^C overnight to aid de-hulling using laboratory rice mill model JGMJ8098, where the husk and dirt were separated by an in-built aspirator. The obtained brown rice was homogenized and sieved through a 1-mm sieve in Foss Cyclotec mill to obtain the flour of each sample for further analysis.

### Sample selection for near infrared reflectance spectroscopy spectra

We have used a novel approach to selecting representative samples based on stratified purposive sampling where the variation in NIR spectral data were based on clustering similar types of accessions. For this, spectral data of 500 samples were normalized using the standard spectra processing method of MSC. Normalized spectra were subjected to hierarchical clustering by Ward’s method and using squared Euclidean distance. Main clusters were separated and further sub-clustered using the same method. Samples from cluster/sub-cluster center and extreme boundary were taken to form a representative set. All accessions were taken where cluster/sub-cluster had up to four members. A set of 180 accessions was selected for generating reference composition data with wet chemistry analysis.

### Near infrared reflectance spectroscopy spectra acquisition

Five gram of intact flour was scanned on FOSS NIRS 6500 spectrometer equipped with Win ISI Project Manager Software version 1.50 to obtain reflectance spectra. The reference cell (white mica) was scanned before each sample scan to ensure accuracy. Ground sample was loaded in the ring cup with an internal diameter of 3.8 cm and pressed slightly to ensure uniform packing. Each sample was scanned 32 times at 400–2,490 nm at 2 nm intervals, and an average spectrum was recorded for further analysis. The reflectance spectra were expressed as Log (1/R), where R is the respective reflectance. Post scanning the moisture content of samples was estimated to be 9.5–11.9% by AOAC 2005 method 934.01 (20).

### Generation of reference data for near infrared reflectance spectroscopy prediction modeling

The total nitrogen percent (%N) was estimated using Foss Tecator 2300 Kjeltec Nitrogen Auto-Analyser, and it was converted into protein percent by %N*5.95 (AOAC 978.02;^21^). Total dietary fiber (TDF) and total starch of brown rice were estimated using Megazyme Kit K-TDFR and K-TSTA as per AOAC 985.29 and AOAC 996.11, respectively, (22). The AC was estimated iodometrically using pure potato amylose for standard curve development (23). The total oil content was estimated in completely moisture-free, dehulled grain using pulsed NMR spectroscopy which is based on the relaxation of protons when kept in an external magnetic field. The instrument Newport Analyzer Oxford 4000 and the standard operating protocol mentioned in the United States Department of Agriculture NMR Handbook were used (24).

### Quality control

All the estimations were carried out in duplicates to ensure the reproducibility of the results. Suitable standards and reagent blanks were used to ensure accuracy where ASFRM-Rice-2 from PT-8 obtained from INMU, Thailand, was used for method validation and check recovery of protein and TDF, while Total starch control kit (K-TSCK) flours viz. wheat starch, high amylose maize starch were used for method validation of starch. Rice reference materials (BCR-465, 466, and 467) obtained from Sigma-Aldrich were tested for method standardization and validation of amylose estimation. The pulsed NMR-based total oil estimation method is validated using ISO10565:1998 and ISO10632:2000 standard for oilseed and their defatted residues. The instrument was calibrated thrice with reference rice bran oil before the estimation to ensure accuracy of the instrument (25).

### Method for obtaining calibration and validation sets

A unique methodology to ensure uniform distribution of diversity in calibration and validation set was applied. The data of wet chemistry analysis of 180 brown rice accessions were arranged in ascending order for each trait and were divided in the ratio of 2:1 for developing calibration and validation sets, respectively, where every third sample was taken out for preparing the validation set. Thus, two-thirds samples (120) constituted the calibration (training) set, and one-third samples (60) formed the validation (test) set for each trait. This assured that both the sets had equal variability in terms of biochemical parameters which lead to the prevention of bias in data subsets.

### Calibration and validation of near infrared reflectance spectroscopy prediction models

Calibration equations were developed on Win ISI Project Manager Software version 1.50 on the Global Equations program using full spectra. The upper limit for the principal components (PCs) was set at 5, and the components required for the development of the equations were automatically calculated by the software. Multivariate analysis was performed by regressing spectral data with laboratory values. Equations were developed by testing PLS, MPLS, and PCR regression methods coupled with SNV-DT and MSC scatter corrections.

The models were developed by performing various mathematical treatments such as “2,4,4,1” “2,6,6,1” “2,8,8,1” “3,4,4,1” “3,6,6,1” “3,8,8,1” “3,10,8,1” “3,12,8,1” “3,14,8,1” “3,16,8,1” “3,16,8,2” “4,6,6,1” “4,8,6,1” “4,8,8,1” “4,12,8,1” “4,16,8,1,” and “4,16,8,2,” where the first digit is the derivative, second is gap, and third and fourth are first and second smoothening, respectively, (26). Coefficient of determination (RSQ), SEC, SD, and SECV were calculated for developing calibration equations. The generated equations were validated over an external sample set, and reference and predicted values were compared under the Monitor Results program of the WIN ISI software. The best-fit equation was considered qualified as prediction model on the basis of external RSQ, RPD, SEP, bias, and slope values.

### Statistical analysis

The reference laboratory and predicted values from validation set were regressed separately to cross-check the RSQ values and were presented in scatter plots using Veusz plotting package software. Further, a paired sample *t*-test using SPSS Version 17 was performed for predicted and laboratory values to ensure that there is no significant difference between their means at a 95% confidence interval.

Apart from RSQ, the accuracy and applicability of prediction models were ascertained by ensuring low SEP and high RPD values (27):


(1) $$\text{SEP}=\sqrt{\frac{\sum_{i=1}^{n}{\left[x_{1}-x_{2}-b\right]}^{2}}{n}}$$

(2) $$\text{RPD}=\frac{\text{SD}}{\text{SEP}}$$

(3) $$\text{Bias}=\sqrt{\frac{\sum_{i=1}^{n}{\left[x_{2}-x_{1}\right]}^{2}}{n}}$$

where n is the number of validated samples, *x*_1_ and *x*_2_ are the predicted and measured values of the *i*^th^ observation, and b is the model bias, respectively. These equations give the uncertainty that can be calculated in predictions.

## Results and discussion

### Biochemical analysis and near infrared reflectance spectra

**FIGURE 1:**
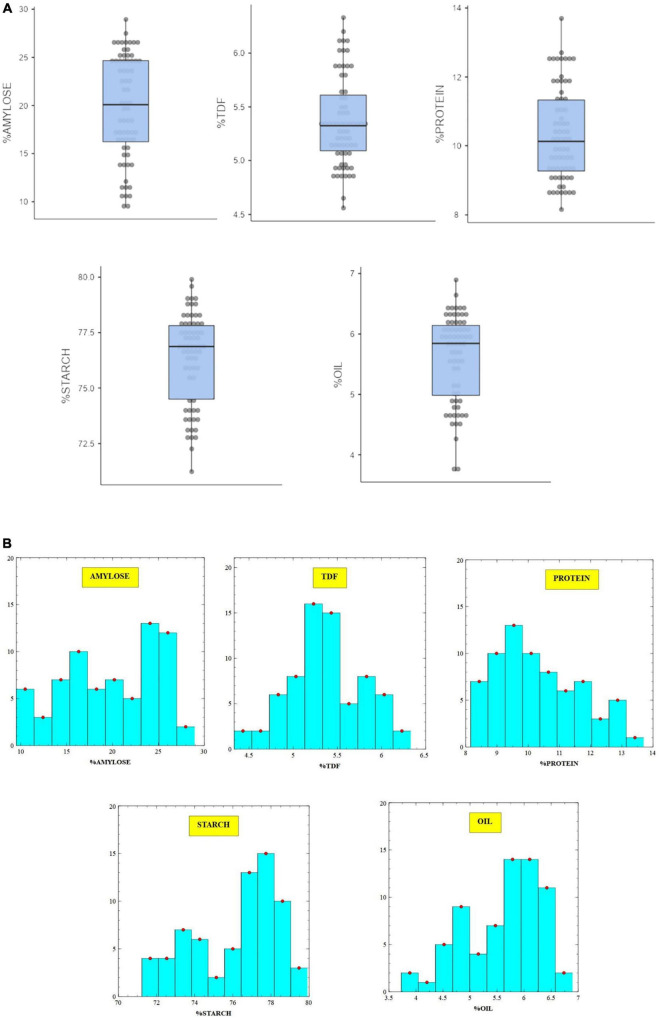
**(A, B)** Variability of biochemical traits in brown rice germplasm through box and whisker plots and histogram.

The stacked biochemical data of nutritional traits, namely, protein, dietary fiber, starch, amylose, and oil obtained by wet chemical analysis are presented as box and whisker plots along with their respective histograms to showcase nearly uniform distribution frequency and extent of variability in the sample set (Figures 1A,B). The results indicate that broad-based equations generated over HCA-clustered data for nutritional traits have the potential to provide more accurate models. The sample selection method also eliminates conventional wet chemistry analysis of large sample sets, which are used to generate reference values (28).

**FIGURE 2:**
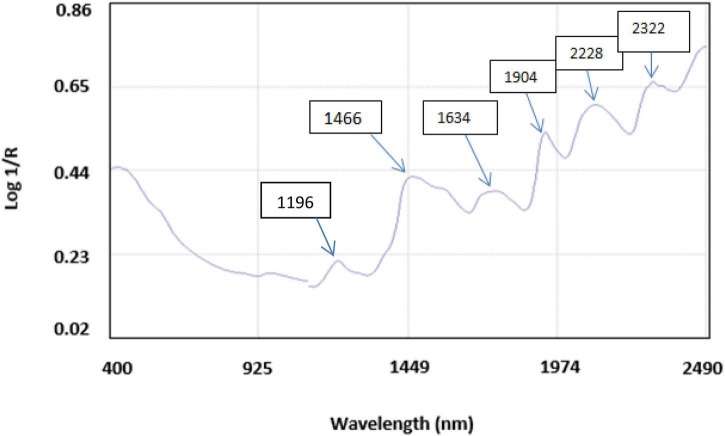
An average NIRS reflectance spectrum of brown rice flour after 32 scans with five major bands corresponding to vibrations due to respective functional groups.

The average NIR reflectance spectrum of 500 accessions of brown rice in the NIR wavelength range of 400–2,490 nm is shown in Figure 2, which gave six major bands at wavelengths 1,196, 1,466, 1,634, 1,904, 2,288, and 2,322 nm. The 1,196-nm band arises due to the C-H second overtone corresponding to aliphatic hydrocarbons. The 1,466-nm band arises due to the O-H functional group from starch, and N-H arises from protein stretching at the first overtone; 1,634-nm band is due to the O-H bending at the second overtone related to water; 1,904-nm band is due to the O-H and C-O bending the second overtone related to starch; 2,288 and 2,322 nm bands are due to the N-H stretching at first overtone relating to protein and C-H bending at second overtone corresponding to oil. Similar bands were observed for PC and protein composition of brown rice flour from Japan (29).

### Regression and calibration

Table 1 shows the mean data of protein, fiber, starch, amylose, and oil estimations in brown rice flour by conventional methodology (*N* = 120), which was used as the reference values to train the model (calibration). All of the three regression algorithms (MPLS, PLS, and PCR) and different pre-processing methods including MSC, weighted MSC, inverted MSC, and SNV-DT were tested (data not shown), and the best results were obtained by the combination of MPLS and SNV-DT.

**TABLE 1:** Traits measured of brown rice flour by conventional methodology.

Calibration				

**Trait**	***N***	**Outliers**	**Range (%)**	**Mean**
Protein	120	7	6.45–14.63	10.35
TDF	120	10	4.43–5.84	5.09
Starch	120	9	65–85.45	75.4
Amylose	120	5	5.23–30.7	23.0
Oil	120	4	3.05–7.00	5.25

N, number of samples.

Results of internal cross-validation of calibration set with developed equation were in good agreement, except for a few outliers (≤10), which may occur because of sample scanning or analytical errors. Such samples generate outlying reflectance spectra and come as a common outlier across the traits, and thus were removed. Hence, a slight shift in the established calibration range after internal cross-validation was observed. Table 2 shows the established calibration range of training set after the removal of outliers, which were 7.33–13.9% for protein (3 PCs), 4.43–5.85% for TDF (3 PCs), 67–80% for starch (5 PCs), 7.56–30.7% for amylose (5 PCs), and 3.05–7% for oil (4 PCs).

**TABLE 2:** Calibration of NIRS models with brown rice flour.

Trait	*N*	Range (%)	Math Treatment	Mean	No. of pcs	RSQ	SLOPE	SD	SEC (V)
Protein	113	7.33–13.9	4,8,8,1	10.50	3	0.782	1.003	1.37	0.663
TDF	110	4.43–5.85	3,16,8,2	5.04	3	0.897	0.954	0.28	0.103
Starch	111	67.0–80.0	4,6,61	75.24	5	0.807	1.012	1.98	0.992
Amylose	115	7.56–30.7	2,8,8,1	24.18	5	0.752	1.024	4.51	2.873
Oil	116	3.05–7.00	4,8,8,1	5.42	4	0.843	1.028	0.74	0.410

N, number of samples; PCs, Principle components; RSQ, coefficient of determination; SD, standard deviation; and SEC(V), Standard error of Cross Validation.

Different combinations of derivative, gap, and smoothening gave the best performance for each trait. Out of many permutations and combinations used, the finalized treatments identified based on performance in external validation were 4,8,8,1 for protein and oil, 3,16,8,2 for dietary fiber, 4,6,6,1 for starch, and 2,8,8,1 for amylose based on high RSQ, low SD, and SEC(V) values. Scattering effects of the sample are usually compensated in the calibration by using only first and second derivatives. Whereas derivatization up to third and fourth order enhanced weak bands that are not prominent in the average spectrum and helped in establishing good regression with reference data as in the case of dietary fiber, starch, and protein. However, the use of higher derivatives also enhances the non-responsive regions of the spectra resulting in over fitting of the models. This was observed in the case of dietary fiber (data not shown); and therefore, to reduce the impact of non-responsive regions, the selection of wavelength segments at constant intervals (gap; up to 16) was used to select responsive segments in spectra that provided better results. Smoothening (S1, S2) was employed to reduce the signal-to-noise ratio in the spectral region due to high-frequency perturbations (30).

### External validation of calibration models

**FIGURE 3:**
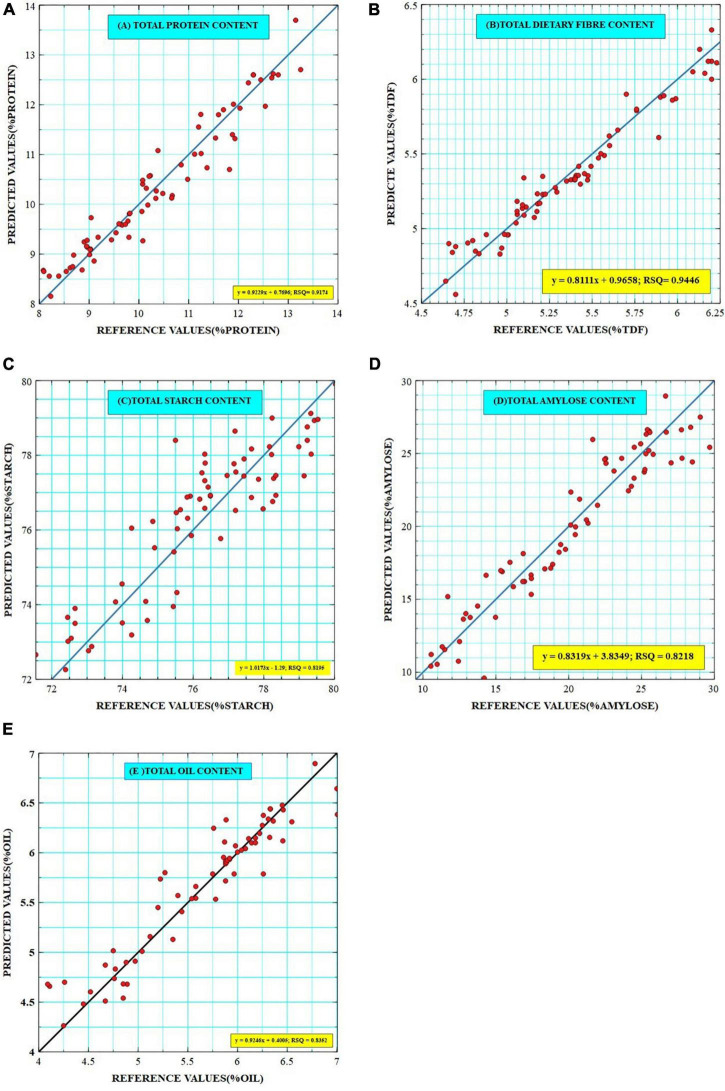
Scatter plots of reference and predicted values of **(A)** Total Protein, **(B)** Total Dietary Fiber, **(C)** Total Starch, **(D)** Total Amylose, and **(E)** Total Oil contents as generated by Veusz software. The key represents a linear equation with RSQ values for each trait.

The generated calibration equations were externally validated over a smaller set of samples which also represented almost the entire range of variability (*N* = 60). Outliers in external validation are often removed to show the high prediction power of developed models but here the external validations were achieved without the removal of outliers to ensure robustness. Based on predicted values, the established validation ranges were 8.15–13.7% for protein [comparable to Bagchi et al. (7)], 4.64–5.55% for TDF [comparable to Longvah et al. (30)], 71.2–78.9% for starch [comparable to Deepa et al. (31)], 9.5–28.94% [wider than that reported by Bagchi et al. (7)] for amylose, and 3.72–6.89% for oil [consistent with Abubakar et al. (32)], which show a good agreement with the calibration ranges (Table 3). The best-fit models were selected on the basis of coefficients of determination such as RSQ, RPD, slope, and bias values. RSQ value defines the correlation between the predicted and reference values around the straight line, which determines the validity and accuracy of prediction. It was observed that high RSQ values were obtained for each trait as 0.945 for dietary fiber, 0.917 for protein, 0.835 for oil, and 0.820 and 0.822 for starch and amylose, respectively. Similar RSQ value of 0.85 for predicting AC of Japanese brown rice has been reported (33). Prediction models have been established for estimating fat content in Chinese brown rice cultivars with RSQ value of 0.80 (34). A similar validation range of 7–12.6% and RSQ of 0.941 was given for the PC of brown rice (9,35). RSQ value of 0.967 was reported for TDF in Korean brown rice (13); however, our study seems to be the first report on NIRS-based prediction models for TDF in Indian brown rice landraces. Previously reported models also have high RSQ values but they are majorly validated on samples bearing value close to the mean, which limits their usability to general market samples and is not suitable for germplasm resources. The regression plots between the reference and the predicted values for protein, fiber, starch, amylose, and oil are given in Figures 3A–E, respectively.

**TABLE 3:** Validation of NIRS models with brown rice flour.

Trait	*N*	%RANGE (CAL)	%RANGE (VAL)	Math Treatment	RSQ	SLOPE	BIAS	SD	SEP	RPD
Protein	60	7.33–13.9	8.15–13.7	4,8,8,1	0.917	0.994	–0.012	1.14	0.329	3.46
TDF	60	4.43–5.85	4.64–5.55	3,16,8,2	0.945	1.164	0.018	0.25	0.069	3.62
Starch	60	67.0–80.0	71.2–78.9	4,6,6,1	0.820	0.806	–0.024	1.73	0.816	2.12
Amylose	60	7.56–30.7	9.50–28.9	2,8,8,1	0.822	0.988	0.120	5.44	2.298	2.36
Oil	60	3.05–7.00	3.72–6.89	4,8,8,1	0.835	0.903	0.025	0.73	0.306	2.39

N, number of samples; RSQ, coefficient of determinations; SD, standard deviation; SEP, standard error of prediction; and RPD, residual prediction deviation.

Apart from high RSQ values, RPD value also governs the prediction accuracy of the models. RPD is defined as the ratio of prediction to standard deviation of reference values, where RPD < 1.0 indicates very poor model/predictions with no recommended use; RPD between 1.0 and 1.4 indicates poor predictions where only high and low values are distinguishable; RPD between 1.4 and 1.8 indicates fair predictions which may be used for assessment and correlation; RPD values between 1.8 and 2.0 indicates good predictions where quantitative predictions are possible; RPD between 2.0 and 2.5 indicates very good, quantitative predictions, and RPD > 2.5 indicates excellent predictions (36). Our results exhibited RPD value > 2 for each trait as for starch (2.12), amylose (2.36), and oil (2.39) indicating their usability in screening large collections and quantitative predictions. RPD value > 3 was observed for protein (3.46) and TDF (3.62) indicating that the model is providing results that are at par with wet chemical analysis. Similar RPD values of 3.76 and 2.42 were observed for PC and AC in Kadus rice from Iran (37), respectively.

The slope represents a change in predicted values with a unit change in reference values. An ideal slope value should be 1, but any value close to 1 would also represent the accuracy of the model. The slope values in our study are 0.994 (protein), 1.16 (TDF), 0.806 (starch), 0.988 (amylose), and 0.903 (oil), which show optimum accuracy. Comparable slope values of 0.995 and 1.011 have been reported for AC and PC (38), respectively.

Biasness is the average of residuals of laboratory and reference values, which also account for prediction accuracy, and should have a value close enough to 0. A negative value of bias relates to underestimation by the model, whereas a positive bias value depicts overestimation by the model (39). The bias values were −0.012 (protein), 0.018 (TDF), −0.024 (starch), 0.120 (amylose), and 0.025 (oil) depicting that the models for protein, starch are slightly underestimating while TDF, amylose, and oil models are slightly overestimating.

Table 4 summarizes that the means of laboratory (reference) data are similar to the analytical (predicted) data. The reference and predicted values for all traits were found to be in great agreement with each other as paired sample *t*-test at a 95% confidence interval gave *p* values > 0.05. The highest *p* value was observed for starch (0.859) followed by protein (0.781), amylose (0.777), oil (0.509), and fiber (0.111). A *p*-value > 0.05 corresponds to the rejection of the hypothesis that the difference in the means of the reference and predicted values are significant confirming the applicability of the models.

**TABLE 4:** Results of paired sample *t*-test between the reference and predicted values.

		Paired differences					*t*-value	DF	*p*-value
		Mean	Std. Deviation	Std. Error Mean	95% Confidence Interval of the Difference				
					Lower	Upper			
Pair 1	Protein – Protein predicted	−0.011949	0.328882	0.042817	−0.097656	0.073758	−0.279	59	0.781
Pair 2	TDF – TDF predicted	0.017550	0.067970	0.010747	−0.004188	0.039288	1.633	59	0.111
Pair 3	Starch – Starch predicted	−0.023895	0.826231	0.134032	−0.295470	0.247681	−0.178	59	0.859
Pair 4	Amylose – Amylose predicted	0.119833	2.297697	0.419500	−0.738141	0.977808	0.286	59	0.777
Pair 5	Oil – Oil Predicted	0.025359	0.305568	0.038196	−0.050969	0.101688	0.664	59	0.509

This predicts that there is no significant difference between the reference and predicted values (at 5% confidence level); t, test statistic; DF, degrees of freedom; and p, probability of attaining results under the null hypothesis.

## Conclusion

The present study was carried out to develop a rapid assessment tool for screening rice germplasm collections and develop NIRS-based prediction models for brown rice flour. The developed models are robust with applicability for a wide range of variability in each trait. The best models were developed for TDF followed by protein, oil, amylose, and starch. Stratified purposive sampling based on NIR spectral data provided nearly uniform distribution of samples for the entire range of variability in each trait, including extreme values which ensured proper learning for training the model. The selection of multiple derivatives and gaps contributed to enhancing the model performance by enabling feature extraction and regression with responsive regions. The developed prediction models are useful for screening vast germplasm collections of rice and categorize them for specific nutritional needs and culinary purposes. In the future, models for whole grains are to be developed, which would be truly non-destructive and of direct use for plant breeders and eliminate the requirement for sample homogenization.

## Data availability statement

The raw data supporting the conclusions of this article will be made available by the authors, without undue reservation.

## Author contributions

RJ: investigation and writing original draft. CJ, HB, and NS: inter-laboratory method validation for generating wet lab data and manuscript review. GH, DN, MA, DS, SS, and ML: selected and provided diverse rice landraces based on agro-morphological traits. RS, SA, and AK: review and supervision. RB: conceptualisation, monitoring, review of results and editing. JCR: coordination, critical review and fine editing. All authors contributed to the article and approved the submitted version.

## Abbreviations

AOAC: association of analytical chemists
SNV-DT: standard normal variate and detrend
MSC: multiplicative scatter correction
PLS: partial least square
PCR: principle component regression
MPLS: modified partial least square
RSQ: coefficient of determination
RPD: ratio of prediction to deviation
SEP(C): standard error of prediction.
